# Analysing tropical elasmobranch blood samples in the field: blood stability during storage and validation of the HemoCue® haemoglobin analyser

**DOI:** 10.1093/conphys/coz081

**Published:** 2019-11-29

**Authors:** Gail D Schwieterman, Ian A Bouyoucos, Kristy Potgieter, Colin A Simpfendorfer, Richard W Brill, Jodie L Rummer

**Affiliations:** 1 Department of Fisheries Science, Virginia Institute of Marine Science, William & Mary, Gloucester Point, VA 23062, USA; 2 Australian Research Council Centre of Excellence for Coral Reef Studies, James Cook University, Townsville, QLD 4811, Australia; 3 PSL Research University, EPHE-UPVD-CNRS, USR 3278 CRIOBE, Université de Perpignan, 58 Avenue Paul Alduy, Perpignan Cedex 66860, France; 4 College of Science and Engineering, James Cook University, Townsville, QLD 4811, Australia; 5 Centre for Sustainable Tropical Fisheries and Aquaculture, College of Science and Engineering, James Cook University, Townsville, QLD 4811, Australia

**Keywords:** haemoglobin concentration, elasmobranch, HemoCue, storage duration, blood, haematocrit

## Abstract

Blood samples collected from wild-caught fishes can provide important information regarding the effects of capture (and thus post-release survival) as well as other stressors. Unfortunately, blood samples often cannot be analysed immediately upon sampling, and blood parameters (e.g. blood oxygen levels and acid–base parameters) are known to change with storage duration due to the metabolic activity of the red blood cells. We obtained blood samples from both untreated and stressed individuals of both blacktip reef shark (*Carcharhinus melanopterus*) and sicklefin lemon shark (*Negaprion acutidens*) to determine the effects of storage duration on blood pH, haematocrit and haemoglobin concentration ([Hb]). We found no significant effects after storage on ice for up to 180 minutes. Moreover, to validate the usability of a HemoCue haemoglobin analyser (a point-of-care device), we compared data from this device to [Hb] determined using the cyanomethaemoglobin method with blood samples from 10 individuals from each of the aforementioned species as well as epaulette shark (*Hemiscyllium ocellatum*). Values from the HemoCue consistently overestimated [Hb], and we therefore developed the necessary correction equations. The correction equations were not statistically different among the three elasmobranch species within the biologically relevant range but did differ from published corrections developed using blood from temperate teleost fishes. Although the HemoCue is useful in field situations, development of species-specific calibration equations may be necessary to ensure the reliability of inter-species comparisons of blood [Hb]. Together, these data should increase confidence in haematological stress indicators in elasmobranch fishes, measurements of which are critical for understanding the impact of anthropogenic stressors on these ecologically important species.

## Teaser

Lay summary

Elasmobranch blood haematological parameters remained stable over 3 hours of storage duration, indicating that they do not need to be measured immediately. The HemoCue haemoglobin analyser can be used to measure haemoglobin concentrations from elasmobranch blood samples with the use of a correction equation.

## Introduction

Elasmobranch populations the world over are being threatened with overfishing, climate change, habitat alteration and shifting trophic dynamics (Dulvy *et al.,* 2014). To effectively conserve these economically and ecologically important species, it is critical to understand how, when, and for how long physiological stress responses occur. Controlled laboratory studies provide invaluable information regarding the mechanisms driving particular responses; however, these studies are frequently difficult to conduct with elasmobranch fishes as capture and transport to shoreside holding tanks often result in high mortality rates (Bernal and Lowe, 2016). Further, experimental results are frequently inapplicable to *in situ* conditions given the variable and multi-stressor nature of real-world conditions (Morash *et al.,* 2018). Complimentary field studies can, therefore, provide essential information regarding the cumulative nature of multiple stressors and can help inform policies that attempt to minimize the impact of stress on wildlife.

Stress has often been quantified from blood parameters including haematocrit (Hct), pH, haemoglobin concentration ([Hb]) and mean corpuscular haemoglobin content (MCHC) (e.g. Wells *et al.,* 1986*,*^1997^; Frick *et al.,* 2010a*,*2010b; Marshall *et al.,* 2012; Johnstone *et al.,* 2017; Whitney *et al.,* 2017). Stress-induced elevations in Hct and [Hb] are assumed to reflect a greater ability to transport oxygen, thereby meeting increased metabolic demands (Wells, 2009). Limitations to this assumption do exist, as increases in Hct (usually accomplished through splenic contraction—a common stress response in teleost fishes; Gollock *et al.,* 2006, Mandic *et al.,* 2009) can increase blood viscosity, thus impeding rapid oxygen transport (Wells and Baldwin, 1990). A change in pH can also indicate stress, with metabolic and respiratory acidosis disrupting homeostasis and potentially reducing blood-oxygen affinity (Bohr effect) (Berenbrink, 2011; Nikinmaa *et al.,* 2019) and maximum blood-oxygen carrying capacity (Root effect) (Verde *et al.,* 2007; Waser, 2011). Red blood cell (RBC) swelling (often indicated by a reduction in MCHC) in both teleost and elasmobranch fishes can accompany reductions in plasma pH (Brill *et al.,* 2008; Chapman and Renshaw, 2009; Rummer *et al.,* 2010).

While these metrics are generally considered to be simple and straightforward, there are difficulties associated with measuring haematological parameters in field settings. Rapid assessment of Hct, pH and [Hb] can be challenging, as each metric requires specific equipment and in the case of [Hb], chemical reagents. These challenges have led to a trend in measuring blood parameters after several minutes or even hours (Clark *et al.,* 2011). As nucleated RBCs are metabolically active, storing blood until a time when it can be measured in a laboratory setting may allow for sufficient respiration to significantly change metabolite concentrations and the partial pressure of carbon dioxide of the plasma, thus shifting pH (Korcock *et al.,* 1988; Clark *et al.,* 2011). This could induce RBC swelling where it was previously absent, changing Hct and [Hb]. As a few recent studies have challenged the assumption that elasmobranch RBCs do not exhibit swelling (Brill *et al.,* 2008; Chapman and Renshaw, 2009), or do not possess the same mechanisms by which teleost fishes are known to swell their RBCs (Rummer *et al.,* 2010), this may be a concern in elasmobranch blood samples.

The cyanmethaemoglobin method of measuring [Hb] requires the use of potassium cyanide-potassium ferricyanide solution (Drabkin’s solution; Dacie and Lewis, 1975) that may be prohibited aboard commercial fishing vessels or at protected field sites because of its acute toxicity. After mixing with Drabkin’s solution, samples must be measured using a spectrophotometer, an instrument that is not easily transported. In this method, Drabkin’s reagent haemolyses the RBCs, and the ferricyanide oxidizes haemoglobin to produce methaemoglobin. This binds to cyanide to form a cyanomethaemoglobin complex, the absorbance of which is used to calculate [Hb]. While it is possible to freeze blood samples for later measurement using the cyanmethaemoglobin method, transporting frozen samples over long distances can be difficult. Point-of-care devices can be used to circumvent these problems (Stoot *et al.,* 2014; Lindholm and Altimiras, 2016). These devices were developed for human medical care, however, and therefore must be validated for use with other vertebrates (Harter *et al.,* 2015; Talwar *et al.,* 2017).

The HemoCue haemoglobin analyser has been used in studies spanning an array of fish species (e.g. Arnold, 2005; Clark *et al.,* 2010; Raby *et al.,* 2015; Ekström *et al.,* 2016; Morash *et al.,* 2016) and has been calibrated for use in teleost blood (Clark *et al.,* 2008; Andrewartha *et al.,* 2016), but has not yet been validated for use with elasmobranch blood. The HemoCue uses cuvettes coated with sodium deoxycholate, which lyses the RBCs, and sodium nitrite, which converts the haemoglobin to methaemoglobin. Sodium azide then complexes with the methaemoglobin to from azidemethaemoglobin, and the absorbance of the sample is measured at 565 and 880 nm. The dual wavelengths are designed to compensate for turbidity in the sample. As the internal calculations have been calibrated for use on human blood, both Clark *et al.* (2008) and Andrewartha *et al.* (2016) found the HemoCue consistently overestimated [Hb] and posited that this was likely due to the fact that human RBCs are anucleated while fish RBCs are nucleated.

While there are many similarities between elasmobranch and teleost blood composition, there are a few differences that could potentially impact the values reported by the HemoCue (Morrison *et al.*, 2015). First, as mentioned before, teleost RBCs are known to swell in response to acidosis while elasmobranch RBCs are not (Rummer *et al.,* 2010, Morrison *et al.*, 2015). Thus, RBC membrane transporters and the mechanisms governing intracellular buffering capacity could be different from the diversity of mechanisms observed in teleost fishes (Wilhelm Filho *et al.,* 1992; Jensen, 2004). Elasmobranch blood also does not exhibit a decline in maximum oxygen carrying capacity under acidosis (Berenbrink *et al.,* 2005), which suggests the structure of elasmobranch haemoglobins is likely to differ from those of teleost fishes (Morrison *et al.*, 2015). As the HemoCue is directly measuring absorbance by the respiratory pigments, this structural change could result in a different correction factor. Finally, elasmobranch fishes tend to have fewer but larger RBCs in circulation when compared to teleost fishes (Wilhelm Filho *et al.,* 1992). The difference in cell density and relative proportion of cellular debris in the lysed sample could also impact absorbance readings.

Here, we field test the stability of elasmobranch blood samples on ice over time and the reliability of the HemoCue haemoglobin analyser to measure [Hb] in elasmobranch blood. With regard to haematological parameter stability over time, we test the null hypotheses that (i) storage duration has no effect on haematological parameters, and (ii) stress has no effect on the impact of storage duration. With regard to the HemoCue validation, we test (i) whether elasmobranch [Hb] values need to be corrected to match values obtained using the Drabkin’s method, and (ii) whether elasmobranch [Hb] values obtained using the HemoCue haemoglobin analyser require the same correction as teleost [Hb] values.

We used blood samples from untreated and stressed juvenile blacktip reef (*Carcharhinus melanopterus*) and sicklefin lemon sharks (*Negaprion acutidens*) to address questions regarding storage duration of elasmobranch blood samples. We used blood from these two species as well as from the epaulette shark (*Hemiscyllium ocellatum*) to correlate haemoglobin values measured by the HemoCue haemoglobin analyser to that measured using the cyanmethaemoglobin method. These three elasmobranch species are common in the tropics and easily maintained in captivity. The species examined in this study are from two disparate orders and thus represent some of the diversity of the elasmobranch group.

## Materials and methods

All capture, handling and experimental protocols were approved by the James Cook University Animal Ethics Committee (A2588 and A2394).

### Blood storage duration

Juvenile blacktip reef and sicklefin lemon sharks were collected in the waters of Moorea, French Polynesia using a gill net (50 m long, 1.5 m tall, 5 cm stretch mesh). Individuals were transported to the Centre de Recherches Insulaires et Observatoire de l’Environnement, where they were maintained in temperature-controlled flow through systems at 28°C as described by Bouyoucos *et al.* (2018). Epaulette sharks were collected by snorkelers who captured the animals by hand near Orpheus Island. Sharks were then transported to the Aquaculture Research Facility Unit at James Cook University. All individuals were given at least 2 weeks to habituate to captivity before being sampled.

To determine the stability of elasmobranch haematological parameters over time, we took 2 ml of blood samples *via* caudal venipuncture using heparinized syringes both from blacktip reef and sicklefin lemon sharks held in captivity (hereafter referred to as “untreated”; *N* = 10 for each species), and those recently captured by gill-net (hereafter referred to as “stressed”; *N* = 10 for each species). Stressed individuals were caught as part of the long-term monitoring program in Moorea and, following capture, were held out of water in brief bouts (<10 minutes combined) while they were weighed, measured, pit-tagged and photographed. Blood samples were taken just prior to release, following handling. No anaesthesia was used in this study.

All blood samples (both from untreated and stressed sharks) were transferred to Eppendorf tubes and maintained in an insulated cooler with ice packs for up to 3 hours. Measurements were taken immediately after blood was transferred to Eppendorf tubes, with pH being the first metric measured, and then after 30, 90 and 180 minutes of storage. Time points were selected to be longer than we typically encountered during the long-term research program in Moorea. We measured haematocrit using microcentrifuge tubes (Globe Scientific, Mahwah, NJ, USA) and in a Zipocrit haematocrit microcentrifuge (LW Scientific, Lawrenceville, GA, USA) for 5 minutes at 11000 rpm. We measured blood pH using a Hanna Instruments HI 99161 pH meter (Hanna Instruments, Woonsocket, RI, USA) calibrated with a two-point calibration with 7.01 and 4.01 (at 25°C) buffer solutions (HI 50004; Hanna Instruments). Calibrations were conducted in ambient lab air temperature (~25°C) following Talwar *et al.* (2017). Stored blood samples were warmed by hand to approximately the same temperature as the first sample, but blood pH was not temperature corrected as the meter has a built-in temperature correction formula. Raw pH readings were corrected using the equation provided by Talwar *et al.* (2017):

(1) \begin{equation*}{\mathrm{pH}}_{\rm corr}=\frac{{\mathrm{pH}}_{\rm Hann}-1.4517}{0.78385}\end{equation*}

where pH_corr_ is the pH standardized to the iStat system, and pH_Hann_ is the raw pH reading provided by the handheld Hann pH meter.

[Hb] was measured using the Drabkin’s methods with a 96-well plate and a Biotek 800 TS spectrophotometer (BioTek Instruments, Inc., Winooski, VT, USA) following the methods described by Arnaud *et al.* (2016). We also calculated MCHC as [Hb]/(Hct/100).

### HemoCue validation

For the HemoCue validation experiments, juvenile blacktip reef (*N* = 10) and sicklefin lemon sharks (*N* = 10) were sampled, as well as epaulette sharks (*N* = 10). Blood draws were conducted on captive, unstressed animals, and were analysed separately from the blood draws described previously. Blood was drawn *via* caudal venipuncture using a heparinized syringe and immediately transferred to an insulated cooler with ice packs. Within 15 minutes, each blood sample was divided among five Eppendorf tubes. Two of these tubes were spun using a benchtop microcentrifuge (5 minutes at 6000 rpm) to separate the plasma. A fraction of this plasma was removed to concentrate the RBCs, and then the Eppendorf tubes were gently vortexed to resuspend the packed RBCs. Additional plasma was added to two of the remaining three Eppendorf tubes to dilute the RBCs, thus creating a range of [Hb] greater than what would be observed in nature (Clark *et al.,* 2008; Andrewartha *et al.,* 2016).

**Figure 1:**
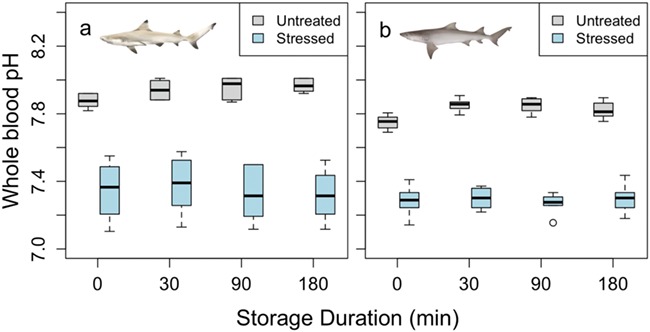
Whole blood pH for both blacktip reef (a) and sicklefin lemon (b) sharks. There was a significant difference between the untreated (*N* = 10) and stressed (*N* = 10) samples in both species (*P* < 0.01), but there was no significant effect of storage duration within species and within treatment

The [Hb] for each sample was measured using both the HemoCue analyser and the traditional cyanomethaemoglobin method. For measurement with the HemoCue, initial tests of incubation times between 30 seconds and 15 minutes suggested that [Hb] remained constant after 9 minutes of incubation in HemoCue cuvettes, rather than the 30 seconds suggested by the manufacturer. This has also been observed with teleost blood (Andrewartha *et al.,* 2016). For the cyanomethaemoglobin method, 7.5 μl of blood was added to 1.5 ml of Drabkin’s and Brij solution, allowed to incubate for at least 5 minutes, then the absorbance at 540 nm was read in quadruplicate in a 96-well plate using a Biotek 800 TS spectrophotometer (BioTek Instruments, Inc., Winooski, VT, USA).

### Statistical analyses

Differences between the different time steps of storage duration, as well as between the two treatment groups, were analysed using a repeated measures linear model, where time step and treatment were fixed effects, and individual and location (either holding tank for untreated individuals, or capture site for stressed individuals) were treated as nested random effects. Analyses were conducted using the nlme package (Pinheiro*, et al.,* 2014) in R (Team RDC, 2019).

For the HemoCue validation, a linear regression was fit to [Hb] measurements made with the HemoCue and cyanmethaemoglobin methods. Slope and intercept vales were then compared to equivalent equations developed for teleost blood (Clark *et al.,* 2008, Andrewartha *et al.,* 2016). Significant differences were determined using the 95% confidence intervals (CIs) around the slope and intercept values. Data were also pooled to generate a global regression.

## Results

### Storage duration

**Figure 2:**
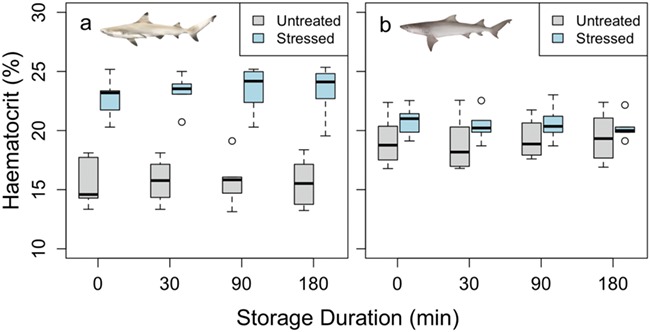
Haematocrit for both blacktip reef (a) and sicklefin lemon (b) sharks. Although there was separation in values between the untreated (*N*−10) and stressed (*N* = 10) samples in blacktip reef shark (*P* = 0.09), this was not evident in the sicklefin lemon shark samples. There was no significant effect of storage duration within species and within treatment

**Figure 3:**
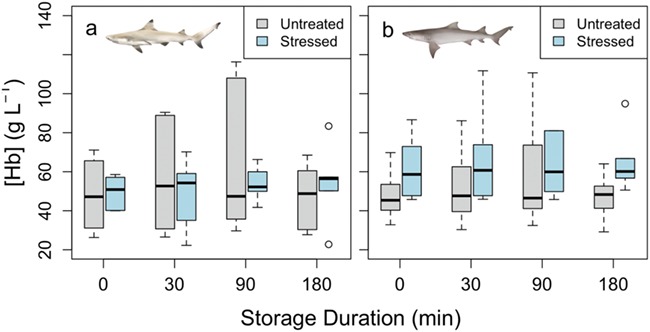
[Hb] for both blacktip reef (a) and sicklefin lemon (b) sharks. There was no significant difference between the untreated (*N* = 10) and stressed (*N* = 10) samples for either species, and there was no significant effect of storage duration within species and within treatment

**Figure 4:**
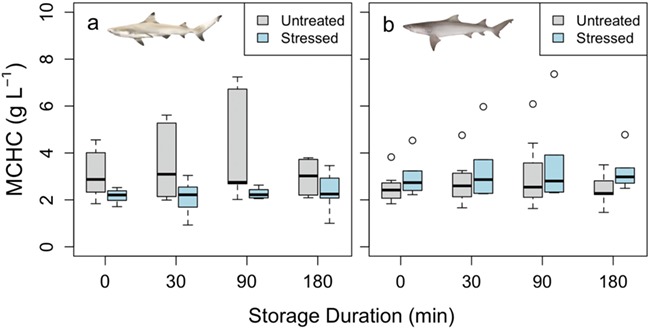
MCHC for both blacktip reef (a) and sicklefin lemon (b) sharks. There was no significant difference between the untreated (*N* = 10) and stressed (*N* = 10) samples in either species, and there was no significant effect of storage duration within species and within treatment

Blood pH was measured at a consistent temperature across species and treatments (24.9 ± 0.2°C, mean ± standard error [SE]). Blood pH was lowered significantly in stressed blacktip reef and sicklefin lemon sharks compared to untreated sharks (*F* = 74.8 and 581, respectively; *P* < 0.05, Fig. 1) with changes from 7.93 ± 0.01 and 7.82 ± 0.01 to 7.34 ± 0.03 and 7.29 ± 0.01 (mean ± SE), respectively. In contrast, Hct values were elevated in stressed blacktip reef sharks from 15.6 ± 0.4 to 23.3 ± 0.3% (mean ± SE), although this was not significant (*F* = 142, *P* = 0.09; Fig. 2). There was no difference in Hct between untreated and stressed lemon shark blood samples (*F* = 0.53, *P* = 0.60; Fig. 2). There were no significant differences in [Hb] or MCHC between the untreated and stressed blood samples of either species (*F* = 0.52, *P* = 0.26 for blacktip reef sharks; *F* = 0.23, *P* = 0.71 for sicklefin lemon sharks). We also found no effect of storage duration on pH, Hct, [Hb] or MCHC (*P* > 0.05; Figs 1–4, respectively) in blood samples from either species, although it should be noted that variability was high in [Hb] measurements, particularly in blacktip shark (Fig. 3).

**Table 1:** Results of the linear regression for each species

Species	Intercept	Slope	*r* ^2^	Source
Blacktip reef shark	−13.08 ± 2.10	1.03 ± 0.03	0.97	This study^a^
Sicklefin lemon shark	−4.10 ± 4.12	0.86 ± 0.05	0.89	This study^b,c^
Epaulette shark	−5.30 ± 1.35	0.91 ± 0.03	0.95	This study^b^
Sockeye salmon	−5.831	0.820		Clark *et al.* 2008 ^c^
Chinook salmon				
Pacific bluefin tuna				
Chub mackerel				
Atlantic salmon	−2.198	0.815	0.98	Andrewartha *et al.* 2016 ^c^

Data are presented as linear model output ± SE. Significance is shown through superscripts on the source.

**Figure 5:**
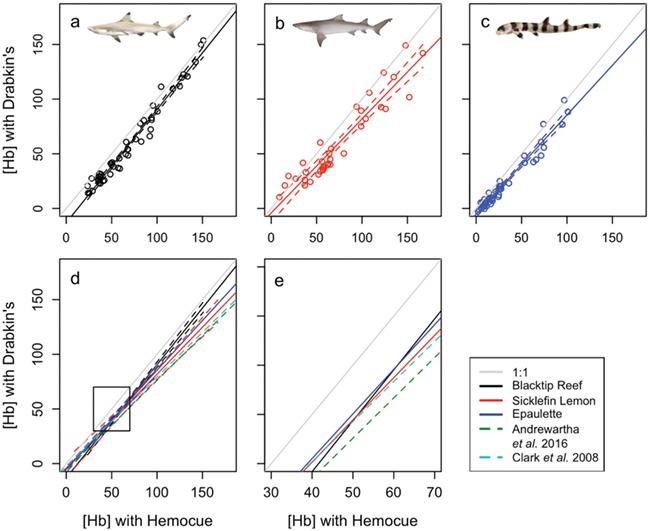
HemoCue validation for (a) blacktip reef, (b) sicklefin lemon and (c) epaulette sharks (*N* = 10 for each species), with raw data in open circles and 95% CIs plotted in the dotted lines. The light grey line represents the 1:1 line in all plots. All three regressions are plotted together in panel (d), along with the published correction equations calculated from teleost fishes. The box in panel (d) represents the zoomed in section represented in panel (e), which shows the intersections over a biologically relevant haemoglobin concentration ([Hb]) range. Here, CIs have been excluded for clarity

**Figure 6:**
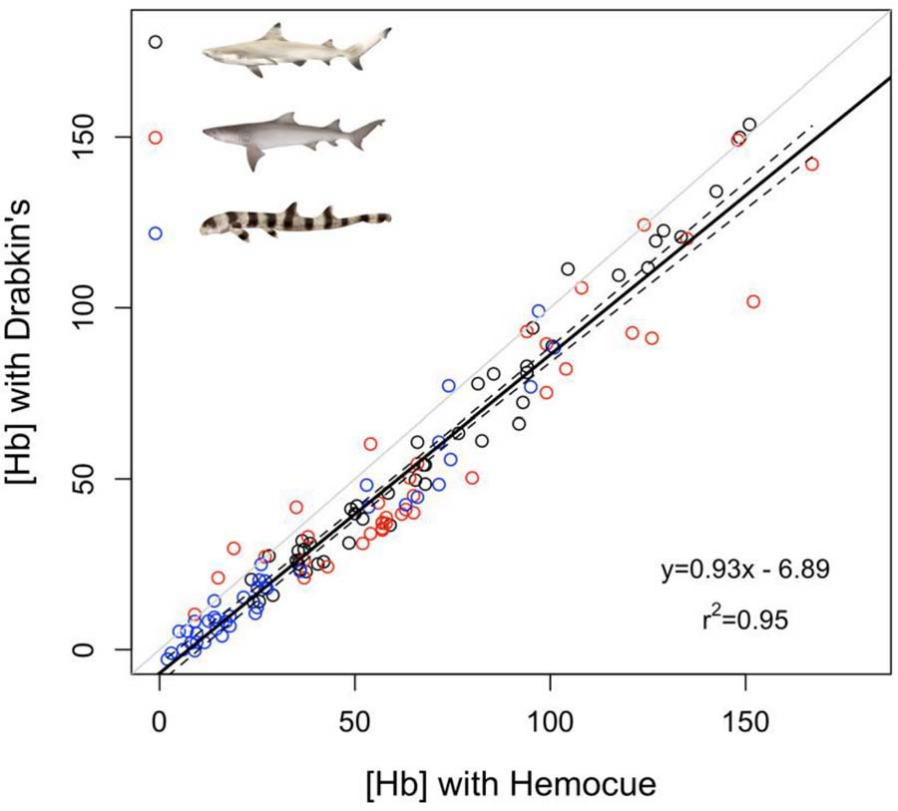
HemoCue validation for blacktip reef, sicklefin lemon and epaulette sharks (*N* = 10 for each species), with raw data in black, red and blue open circles, respectively. 95% CIs are plotted in the dotted lines. The light grey line represents the 1:1 line

### HemoCue validation

The relationship between [Hb] measured by the HemoCue and cyanmethaemoglobin methods was linear for all three species, (Table 1; Fig. 5), although the regression parameters were species-specific. Blacktip reef sharks had significantly different correction equations when compared to the sicklefin lemon shark (*t* = −3.24, *P* < 0.01) and the epaulette shark (*t* = −2.99, *P* < 0.01), but there was no significant difference between the sicklefin lemon shark regression and that of epaulette shark (*t* = 0.75, *P* = 0.45). When compared individually, both the blacktip reef and the epaulette shark exhibited significantly different equations to those published by Clark *et al.* (2008) and Andrewartha *et al.* (2016) as determined using 95% CI. It is important to note, however, that, when examining the intersection points between these regression lines (Fig. 5e), it is evident that they intersect within a biologically relevant [Hb] range. Given this high level of overlap in the biologically relevant range, we recommend using the following correction equation from all three species in this study

(2) \begin{equation*}{\left[\mathrm{Hb}\right]}_{\mathrm{corr}}=\left({\left[\mathrm{Hb}\right]}_{\mathrm{raw}}\times 0.93\right)-6.89\end{equation*}

where [Hb]_corr_ is the corrected value that is equivalent to values derived from the cyanomethaemoglobin method, and [Hb]_raw_ is the value provided by the HemoCue (Fig. 6).

## Discussion

Through a systematic validation of field-based physiological methodologies, we can increase both the confidence and reliability of field-based physiological assessments, thus providing tools to facilitate much-needed research regarding *in situ* stress levels and responses. Here, we evaluated two different aspects of elasmobranch haematological analyses and found both were valid.

### Blood storage duration

The lack of significant differences in measured parameters between blood measured immediately after sampling and after 3 hours of cold storage suggests physiologically relevant parameters remain stable over this interval, potentially driven by metabolic suppression due to cool storage temperatures. If this is the case, equivalent suppression of metabolic activity in nucleated fish RBCs *via* a reduction in temperature may not be effective for other species. The two tropical species examined in this part of the study were caught at temperatures between 26 and 34°C and maintained in the laboratory at 28°C. Cold-water fish species will have enzymes optimized to function at low temperatures; therefore storing blood at low temperatures may not suppress metabolic activity as we observed (Tamburrini *et al.,* 1992). We also were concerned that storage in non-airtight containers with large headspaces could affect measured parameters, but the lack of significant differences between parameters measured immediately and those measured after 3 hours suggests air exposure did not affect the measured parameters of these samples.

In agreement with results from Nikinmaa *et al.* (1984), and Jensen (1987), we also observed differences in blood pH between blood samples from untreated and stressed individuals, which suggests that this metric can be used to assess acute stress in elasmobranch fishes. Morgan and Iwama (2011) have posited that other metrics such as blood glucose, [Cl^−^] and [Na^+^] are reliable predictors of acute stress, and Bouyoucos *et al.* (2018) found that lactate also was significantly different between stressed and untreated blacktip reef and sicklefin lemon sharks. Here, we did not observe significant differences in any of the blood parameters we measured from untreated and stressed sharks besides pH. We did observe higher Hct in blood samples from stressed backtip reef sharks relative to untreated animals. This, coupled with the lack of significant changes in MCHC, suggests this species may have undergone mild RBC swelling, increased circulating RBCs or decreased plasma, as was observed in sandbar shark (*C. plumbeus*) (Brill *et al.,* 2008). The species-specific responses observed lead us to advocate for the use of multiple haematological parameters when assessing stress responses in elasmobranch fishes.

We did see substantial variability, particularly in our untreated individuals. It is generally accepted that elasmobranch blood is stable following the handling associated with caudal venipuncture (Cooper and Morris, 1998; Brill *et al.,* 2008). We therefore attribute this to the fact that multiple individuals were maintained together in each holding tank, and we were unable to quantify the stress of other individuals’ capture. Other sources of individual variation could come from differences in the handling stress, the time taken to obtain blood, and whether the blood was obtained from venous or arterial vessels.

### HemoCue validation

As hypothesized, regression line fit for each of the three species did not fall on the 1:1 line, following the findings for teleost fishes (Clark *et al.,* 2008; Andrewartha *et al.,* 2016). However, the regression lines for both epaulette and blacktip reef sharks were significantly different than these published values. The correspondence between the published equations and the sicklefin lemon shark regression line may be attributed to the large scatter around the regression line and thus wider CIs. The overlap of the intersection points between the three regressions calculated here suggests that, while these equations are statistically different, the regression equations are not actually species-specific. This should promote confidence in the use of published correction factors when using the HemoCue, despite the lack of a mechanistic understanding of what may be driving the overestimation in raw HemoCue values from fish blood compared to those obtained with human blood. Work on the molecular diversity of haemoglobin isomers present in fishes would be necessary to determine if the ratio of absorbance to [Hb] is different among fish species and between fishes and human blood (Morrison *et al.*, 2015, Andrewartha *et al.,* 2016).

Together, these results should enable researchers to collect important and informative haematological indicators of stress in education settings (e.g. experiential learning camps for lower school students), aboard commercial and research vessels and in remote or protected areas. Our results suggest that haematological indicators of stress in elasmobranch fishes can be collected if blood samples can be analysed within 3 hours after sampling. It remains unknown, however, if longer storage is likewise possible. To evaluate stress, we recommend measuring a suite of haematological parameters, as we did observe species-specific responses in Hct that may be indicative of RBC swelling. Further, there are other stress indicators in the blood that were not measured here but can provide valuable information regarding physiological status (Renshaw *et al.,* 2012). We also demonstrate the reliability of the HemoCue for measuring elasmobranch [Hb], although we note the use of a correction factor (Fig. 6; Eq. (2)) is necessary to obtain accurate results. By helping to validate both the storage of blood over hours and the use of handheld point-of-care devices, we hope to provide tools that enable more field measurements of stress and provide tools for field researchers seeking to collect information essential for the conservation and effective management of these ecologically and economically important species.
